# Functional Chitosan-Interpenetrating Networks: Next Generation Super-Adsorbents for Biomedical Applications

**DOI:** 10.3390/polym18111282

**Published:** 2026-05-23

**Authors:** Khushi Verma, Lalita Chopra, Carlo Santulli

**Affiliations:** 1 Department of Chemistry, University Institute of Sciences (UIS), Chandigarh University, Gharuan, Punjab 140413, India; khushiverma1348@gmail.com; 2 School of Science and Technology, University of Camerino, 62032 Camerino, Italy

**Keywords:** chitosan, interpenetrating networks, super-adsorbents, biomedical applications

## Abstract

Chitosan-based interpenetrating networks (IPNs) have become highly attractive as advanced super-adsorbent materials due to their ability to combine a high density of functional adsorption sites with enhanced structural stability under physiological conditions. While chitosan offers intrinsic advantages such as biocompatibility, biodegradability, and chemical functionality, its adsorption efficiency, mechanical strength, and long-term stability may offer limited performance in complex biomedical environments. The formation of interpenetrating networks provides an effective strategy to overcome these limitations by interlacing chitosan with other polymer networks, resulting in a synergistic enhancement of physicochemical and adsorption properties. The formation of chitosan-based IPNs offers tunable control of network structure, porosity, swelling behaviour, and adsorption kinetics, which in turn results in enhanced retention and controlled interaction of drugs, biomolecules, toxins, and other therapeutic agents. Variations in polymer composition, crosslinking density, and network interactions further facilitate the controlled tailoring of adsorption properties for targeted biomedical applications. This review presents a comprehensive and critical assessment of recent progress in the fabrication, functionalization, and structure–property relationships of chitosan-based IPNs, with a main emphasis on their super-adsorbent behaviour. Furthermore, this review highlights key biomedical applications of IPNs, including controlled drug delivery, wound healing systems, tissue engineering scaffolds, detoxification platforms, and biosensing devices. Current issues in scalability, stability, and clinical translation are discussed, as well as future perspectives that highlight the potential of chitosan-based IPNs as high-performance, sustainable super-adsorbent materials for advanced biomedical technologies.

## 1. Introduction

Adsorption-based systems have become vital in the biomedical field due to their ability to regulate molecular interactions, manage mass transport and specifically capture biologically significant species even in very complicated physiological conditions. Adsorption is a key factor determining the efficiency, safety, and functionality of various biomedical devices, including controlled drug delivery systems, detoxification platforms, wound dressings, biosorption membranes, and diagnostic interfaces [1]. The growing demand for precision medicine, minimally invasive treatments, and sustainable biomaterials has driven the need for improved adsorbents that perform effectively in biological settings. In biomedical applications, adsorption is a multifaceted process that incorporates electrostatic interactions, hydrogen bonding, coordination chemistry, and diffusion-controlled processes [2]. In drug delivery systems, adsorption is a key determinant of drug-loading efficiency, bioavailability, and release kinetics. The selective adsorption of toxins, metabolites and inflammatory mediators plays a critical role in the therapeutic effectiveness of detoxification and blood purification therapeutics [3]. Similarly, wound healing materials rely on adsorption to control exudate release, retain growth factors, and prevent microbial colonization, whereas bio-separation platforms use adsorption to selectively collect proteins, enzymes, nucleic acids, and cells [4]. Although these systems’ functional performance is determined by their adsorption characteristics, their significance in biomedical applications originates from their capacity to control drug release, eliminate toxic biomolecules, promote tissue regeneration and facilitate biosensing. Therefore, designing materials that are therapeutically relevant requires an understanding of adsorption in a biological context. Given this broad relevance, the development of high-performance biomedical adsorbents has become a prominent research topic in polymer science, biomaterials engineering and translational medicine. Numerous studies have reported that adsorbent-based systems play an important role in biomedical applications. For example, Iqbal et al. [5] demonstrated that chitosan-based materials have a high adsorption capacity and great potential for biomedical applications due to their tunability and good biocompatibility. Similarly, Cárdenas et al. [6] outlined the role of modern chitosan-based biopolymer systems in drug delivery, wound healing, and biosorption, further confirming that adsorption plays an important role in modern biomedical technologies.

However, despite great advances in the development of adsorption-based materials, traditional adsorbents still face major challenges when they are used in the biomedical domain. Activated carbon, inorganic oxides and synthetic resins for instance are highly efficient for the remediation of the environment but they also come with a set of issues such as poor biocompatibility, limited biodegradability, non-specific adsorption behaviour and difficulties in regeneration and long-term stability [7]. These issues become even more critical in physiological systems due to the complexity of biological fluids, the presence of competing species and strict safety requirements. Consequently, a shift has been observed towards the development of biopolymer-based adsorbents that are more compatible with living systems and whose physicochemical properties can be easily tailored. These limitations were reported by Ying Yu et al. [8], who outlined that the traditional adsorbents are not only unstable but also hardly selective and non-biodegradable under physiological conditions, making it indispensable to develop new bio-based materials.

In this area, natural polymers have been the focus of attention because they are easily renewable and biodegradable and also contain functional groups that facilitate interactions with biological molecules in many ways [9]. Among different biopolymers, chitosan has emerged as one of the potential candidates for biomedical adsorption purposes. Chitosan is a cationic polysaccharide derived from chitin and has reactive amino and hydroxyl groups on its backbone [10]. As a result, chitosan can effectively adsorb a wide variety of biomolecules, metal ions and organic compounds through different mechanisms such as electrostatic attraction, hydrogen bonding and chelation, due to the presence of these functional groups. In addition to adsorption, chitosan has biocompatible, biodegradable and antimicrobial properties which make it suitable for a wide variety of applications, such as drug delivery, wound healing, tissue engineering and biosensing [11]. Muqadas Rafiq et al. [12] and Wu et al. [13] respectively published comprehensive studies on chitosan-based drug delivery and anticancer systems. The authors also highlighted the various important biological functions of chitosan, such as its efficient biodegradation, microbial killing capacity and easily adjustable adsorption features. However, the use of pristine chitosan in high-performance adsorption is challenged by several fundamental weaknesses. Chitosan has a relatively low mechanical strength and a limited surface area, and it is unstable in acidic environments; these factors degrade its performance in harsh biomedical environments [14]. Additionally, its adsorptive performance is highly dependent on pH and other environmental variables, which limits its use in complex systems. To overcome these disadvantages, new methods for enhancing the structural stability, adsorption competence, and effective functionalities of chitosan-based materials have been developed. Different chemical methods, including chemical functionalization, graft copolymerization, crosslinking, and making composites, etc., have been used to modify chitosan and enhance its properties [15]. Among these approaches, the formation of interpenetrating polymer networks (IPNs) has emerged as the most prominent method. IPNs are composed of two or more polymer networks that are physically entangled at the molecular level without covalent bonding between them, resulting in a 3D structure with high mechanical strength and stability, along with the possibility to alter functions [16,17]. This special structure allows the combination of various properties of different polymers, enabling the design of materials with enhanced performance compared to single-polymer systems. Recently, researchers have demonstrated the emergence of IPN-based systems. Komankova et al. [18] made a study on polyacrylonitrile–chitosan IPN scaffolds and found that the hybrids were mechanically stronger, more stable and had a better adsorption efficiency in biomedical environments, hence indicating the potential of IPNs for use as advanced multifunctional materials.

Despite these positive improvements, some constraints such as mass manufacturing, repeatability, long-term storage and regulations remain important barriers to the widespread use of chitosan-based IPNs in clinical and industrial contexts. Variations in raw material quality, complex synthesis pathways, and the toxicity of certain crosslinking agents are issues that must be addressed in detail to simplify processes and standardize production. Therefore, it is imperative to continue research efforts in order to develop highly efficient, eco-friendly, and low-cost materials that will help bridge the gap between academic research and practical applications. These issues were also brought up by El-Araby et al. [19] who pointed out the problems with scalability, toxicological risks, and the need for standardized synthesis protocols for effective clinical implementation. The present review paper summarizes functional chitosan-based interpenetrating polymer networks as next-generation super-adsorbents for biomedical applications. The review discusses the fundamentals of chitosan, the classification and concept of IPNs, different ways of functionalization and synthesis methods, as well as the mechanisms which cause adsorption behaviour. Furthermore, the paper highlights recent developments in biomedical applications, along with current challenges and future directions, outlining the potential of these materials to enhance modern biomedical technologies. Despite extensive research on chitosan and its derivatives, the existing literature focuses on individual modification strategies, composite formation or general biomedical applications rather than providing an integrated understanding of their adsorption behaviour in relation to structural design. In particular, limited attention has been paid to the relationship between interpenetrating network design and super-adsorbent performance in biological conditions. This study seeks to fill these gaps by offering a thorough and focused investigation of chitosan-based interpenetrating polymer networks with a particular emphasis on their super-adsorbent behaviour in biomedical applications. Unlike traditional reviews, this study focuses on the interaction between network structure, functionalization tactics and adsorption processes and analyzes how these elements impact performance in drug delivery, detoxification, bio-separation and biosensing systems. This study provides a more targeted viewpoint on the rational design of high-performance chitosan-based biomaterials by combining structure–property relationships with application-specific parameters.

After illustrating the chemical structure and significance of chitosan (Section 2), and the meaning of interpenetrating networks (Section 3), how their realization is completed using chitosan is clarified (Section 4), while in Section 5, the synthesis routes are illustrated (Section 5). A fundamental characteristic of networked chitosan structures is their super-absorbent behaviour, described in Section 6. This encourages biomedical applications (Section 7), in which chitosan competes with other polymers, as in Section 8. Finally, a global perspective on present and future applications is given by illustrating advantages and challenges, which is the objective of Section 9.

## 2. Chitosan

**Figure 1:**
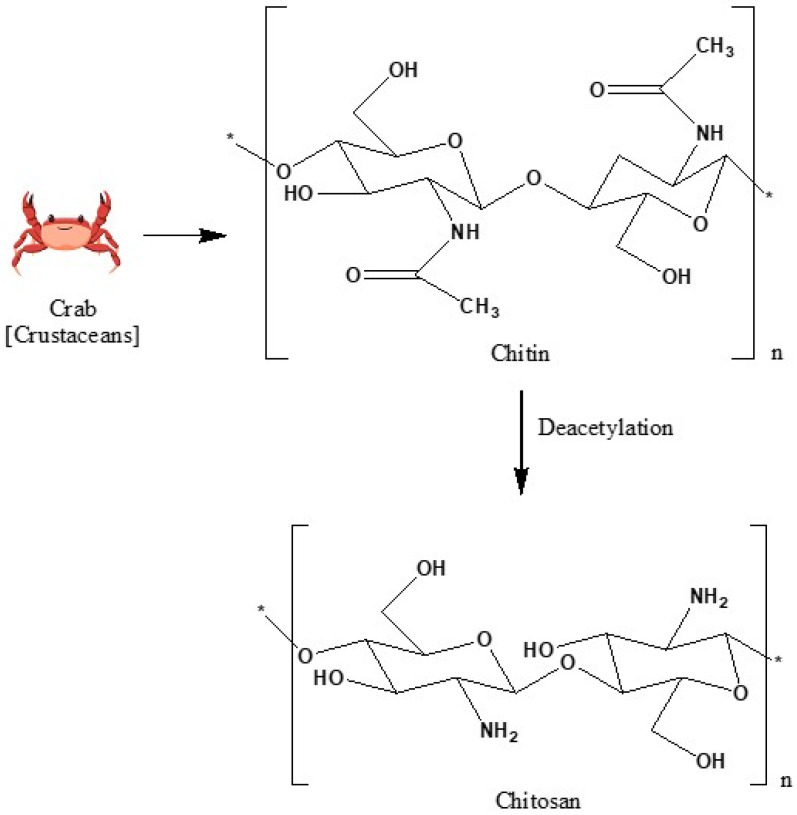
Structure of chitosan.

Chitosan is a naturally occurring biopolymer with the chemical formula (C_6_H_11_NO_4_). It is composed of two units: a deacetylated unit of β-(1→4)-linked D-glucosamine having formula (C_6_H_11_NO_4_) and an acetylated unit of N-acetyl-D-glucosamine (C_8_H_13_NO_5_) [20]. It is obtained from a natural biopolymer, chitin, which is present in the shells of crustaceans like crabs, shrimps, etc. [21]. Chitosan is produced by the deacetylation of chitin using sodium hydroxide as a reagent and water as a solvent [22]. It can be assembled into different forms, such as flakes, beads, powders, membranes, nanoparticles, gels, and sponges [23]. The chemical structure of chitosan is characterized by a high density of primary amino groups (-NH_2_) at the C-2 position and hydroxyl groups (-OH) at the C-3 and C-6 sites of the glucopyranose ring, as reported in Figure 1 [23]. These functional groups play a critical role in influencing the reactivity and adsorption behaviour of chitosan. The amino groups serve as the primary reactive sites accounting for chitosan’s cationic character in acidic conditions and participate in electrostatic interactions, hydrogen bonding and coordinate with metal ions. Hydroxyl groups, although less reactive than amino groups, contribute to hydrogen bonding and increase attraction for polar organic molecules [24]. The degree of acetylation greatly influences the relative abundance of these functional groups, which impacts the chemical and adsorption properties of chitosan [25].

### 2.1. Physicochemical Properties Relevant to Adsorption

The efficiency of chitosan as an adsorbent is mainly influenced by its physicochemical properties, which play an important role in both environmental and biomedical domains. The presence of amine and hydroxyl functional groups on the chitosan polymer chain serves as the active sites for adsorbate binding. Salehi et al. elucidated that these functional groups facilitate adsorption through electrostatic interactions, hydrogen bonding, chelation, and coordination mechanisms, making chitosan an excellent binding agent for metal ions, biomolecules, drugs, etc. [26]. Similarly, Vakili showed that amino groups play an important role in the adsorption process, especially in the removal of dyes, where their capability to engage in electrostatic interactions and complexation gives rise to a very strong attraction to various adsorbates [27,28]. Physicochemical parameters, such as the degree of deacetylation (DD), which controls the number of free amino groups, were extensively reviewed by Rinaudo [29], highlighting their impact on interaction capacity and adsorption behaviour. Consistent with this observation, Wang et al. [30] reported that increased availability of active amino sites improves adsorption effectiveness through surface complexation. The pH-responsive adsorption behaviour of chitosan was detailed by Guibal [25], who reported that changes in pH solution alter the ionization state of amino groups, thereby affecting the binding interactions. Such physicochemical responsiveness has been effectively used in biomedical applications, where pH-dependent drug-loading and unloading behaviour were demonstrated by Dash [23]. Moreover, modification-induced improvements in surface properties and structure stability have been reported to enhance adsorption efficiency and reusability, enabling the effective use of chitosan-based materials in biomedical adsorption applications, including drug delivery and bioactive molecule immobilization.

### 2.2. Biodegradability, Biocompatibility and Bioactivity

Chitosan has been widely investigated for biomedical applications due to its versatile characteristics, including biocompatibility, biodegradability, and intrinsic bioactivity. Several in vitro and in vivo studies have shown that chitosan is non-toxic and well-tolerated by biological tissues, enabling its safe use in drug delivery systems, tissue engineering, and wound healing applications [31,32]. Enzymatic degradation of chitosan under physiological conditions mainly occurs by lysozyme-mediated hydrolysis, resulting in biocompatible oligomers and glucosamine that can be easily metabolized or excreted [33]. Besides its biodegradability, chitosan has intrinsic bioactivity that has been mainly attributed to the electrostatic interactions of its protonated amino groups with the negatively charged microbial membranes, causing the rupture of the membranes and the suppression of microbial growth [34,35]. These combined properties of chitosan make it a very attractive and versatile biomaterial for advanced biomedical applications.

### 2.3. Limitations of As-Received Chitosan as an Adsorbent

In its pure form, chitosan suffers from several intrinsic limitations, despite its desirable functional groups, which hinder its use as an efficient adsorbent. Kyzas and Bikaris [36] highlighted that chitosan in its native form often exhibits a low surface area and limited porosity, which restricts its access to active adsorption sites compared to modified chitosan. The low surface area of pristine chitosan was quantitatively confirmed by BET analysis, showing that unmodified chitosan beads exhibit a surface area as low as 0.708 m^2^/g whereas nanoparticle-incorporated chitosan composites, such as FeO/TiO_2_ -modified chitosan beads, achieve a surface area of up to 27.13 m^2^/g representing a nearly 38-fold improvement in accessible adsorption surface area [37]. Under acidic conditions, bare chitosan exhibits limited mechanical strength and poor stability, as the protonation of amino groups causes swelling and partial solubilization, thereby reducing reusability, as reported by Maslamani et al. [38]. Furthermore, Wang and Zhuang [39] emphasized that the adsorption behaviour of chitosan is extremely pH-dependent, as variations in the protonation state significantly alter surface charge density and binding interactions. Additionally, Crini and Lichtfouse critically reviewed the occurrence of bare chitosan beads demonstrating poor resistance during regeneration cycles and restricted selectivity in complex systems due to competing adsorption effects [40]. Likewise, Santos et al. [41] reported that unmodified chitosan exhibits structural fragility and limited long-term stability in aqueous environments, thereby limiting its large-scale applicability without chemical reinforcement.

## 3. Interpenetrating Polymer Networks (IPNs)

### 3.1. Concepts and Classification

**Figure 2:**
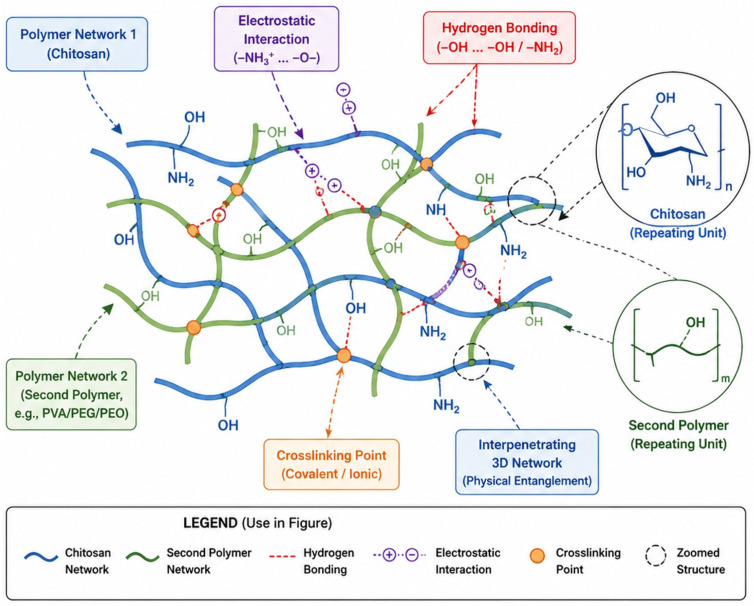
Typical interpenetrated network.

The concept of IPNs originated in 1914, and the first IPN was invented by Alysworth. Miller applied the word IPN to these structures for the first time in the 1960s during his study of polystyrene networks [42]. Interpenetrating polymer networks are materials consisting of two or more polymer networks synthesized and crosslinked so that they become physically intertwined on a molecular scale, but not covalently bonded. Unlike ordinary polymer blends, IPNs have a three-dimensional, interlocked network structure in which one polymer network is produced in the immediate presence of another, resulting in a persistent entanglement that prevents macroscopic phase separation without breaking chemical bonds [17]. This unique structural arrangement allows for the integration of complementary physicochemical features from each constituent polymer into a single material system. The main characteristic feature of IPNs is the close interpenetration of the networks, as depicted in Figure 2, which results in significantly enhanced mechanical strength, structural stability and resistance to solvent-induced deformation compared with single-polymer systems [43].

#### 3.1.1. Full IPNs, Semi-IPNs and Pseudo-IPNs

Interpenetrating polymer network systems are classified based on the nature and extent of network formation in the material. Full IPNs are formed when two or more polymers are synthesized in such a way that each network is chemically crosslinked and permanently interlaced with the other. In this structure, the networks are entangled at the molecular level without covalent bonds between them, leading to combined properties from each polymer while maintaining network integrity [44]. Full IPNs have been widely explored in hydrogel systems where structural cohesiveness and swelling behaviour are critical, such as in drug delivery scaffolds. In contrast, s-IPNs consist of one crosslinked polymer network interpenetrated by a second polymer that remains linear or branched without forming an independent crosslinked network. Here, the non-crosslinked polymer chains are simply physically entangled within the 3D network of the crosslinked component, allowing the modification of swelling behaviour, diffusion characteristics, and drug release patterns. s-IPNs are particularly convenient in biomedical hydrogels where the inclusion of a linear polymer phase allows for increased flexibility, adjustable permeability and controlled therapeutic release while retaining the structural integrity of the primary network [45]. Pseudo-IPNs, also known as physically interpenetrated systems, are formed when the interlaced polymers interact primarily through non-covalent physical interactions such as hydrogen bonding, ionic interaction, or Van der Waals forces. In pseudo-IPNs, the components are not completely crosslinked; network interpenetration is achieved instead by strong secondary interactions. These systems are especially valuable in responsive biomedical hydrogels where reversible interactions (thermal, pH-responsiveness) are needed for stimuli-controlled behaviour. Pseudo-IPNs provide flexibility and responsiveness useful for cell encapsulation, wound dressing, and smart drug delivery matrices.

#### 3.1.2. Advantages over Single-Polymer Network Systems

Interpenetrating networks exhibit various advantages over single-polymer network systems because of the coexistence of two or more polymer components within the same matrix, which allows for the integration of excellent properties. In contrast to individual polymer networks the presence of multiple interlocked polymer phases improves structural integrity, reduces polymer chain mobility and increases resistance to environmental stress. This molecular-level interpenetration increases chemical resistance, thermal stability and solvent resistance by limiting polymer relaxation and dissolution behaviour [46]. In IPNs, one polymer may provide mechanical strength while contributing to stimuli responsiveness, swelling behaviour, or biological functionality, resulting in a multifunctional material with improved performance. Another significant advantage of IPNs is their ability to overcome thermodynamic incompatibility between polymer components through physical entanglement of networks formed during synthesis. This intertwining structure prevents macroscopic phase separation and ensures a uniform distribution of polymer phases, thereby improving structural stability and long-term performance [47]. Additionally, IPNs provide tunable physicochemical properties such as controlled swelling, permeability, and diffusion behaviour, making them particularly valuable in biomedical applications. The interpenetrated network structure allows for improved regulation of mass transfer compared to single-polymer networks [48]. However, these benefits are more noticeable when compared to pure chitosan. Due to the presence of interlocked polymer networks and increased crosslinking density, IPN-based systems show improved tensile strength, elasticity and resistance to deformation. In contrast, native chitosan usually shows limited mechanical strength and decreased structural stability under hydrated conditions [49]. A chitosan/polyacrylic acid/polyacrylamide hydrogel crosslinked with Zr^4+^ ions exhibited a 159% increase in tensile stress rising from 0.37 MPa to 0.96 MPa, compared to the non-crosslinked system which clearly demonstrates how interpenetrating network formation confers substantial mechanical reinforcement over single-component chitosan systems. This clearly shows that the improvement in characteristics is due to structural alteration rather than intrinsic material behaviour.

### 3.2. IPNs in Biomedical Materials

#### 3.2.1. Design Principles for Biomedical IPNs

The design of interpenetrating polymer networks for biomedical applications focuses on obtaining regulated physicochemical properties, biological compatibility and functional performance under physiological conditions. Biomedical IPNs are often produced by integrating structurally stable polymer networks that provide definite biological or stimuli-responsive features like biodegradability, biocompatibility, bioactivity or environmental sensitivity. This strategy allows for fine modulation of swelling behaviour, degradation kinetics and diffusion characteristics, which are critical for drug delivery systems, wound healing materials and tissue engineering scaffolds, etc. [50]. The selection of suitable polymer components and crosslinking mechanisms is a crucial design aspect to enable homogeneous network formation and prevent macrophase separation. The interpenetrated structure enables the separate control of mechanical integrity and biological functioning, which improves the performance of biomedical devices. Additionally, the incorporation of natural polymers, such as chitosan, gelatin or polysaccharides, improves cellular interaction, biocompatibility, and biodegradability, thereby enhancing their adaptability to biomedical applications [51].

#### 3.2.2. Role of Network Interpenetration in Property Enhancement

Gaharwar et al. [52] reported that polymer network interpenetration leads to higher functional performance of biomedical polymer systems by forming a physically interlocked structure, which limits the mobility of polymer chains and stabilizes the matrix under aqueous and physiological conditions. The authors further demonstrated that such molecular-level interlocking increases dissolving resistance and allows for better control of swelling behaviour and molecular transport when compared to a single-polymer network. Similarly, Loh et al. [53] suggested that IPN structures promote synergistic interactions between the constituent polymers, yielding improved control of the diffusion pathways of therapeutic agents and an increase in structural stability during long-term use. Moreover, they pointed out that the existence of interpenetrated networks enhances surface properties and biological interactions, thus facilitating cell adhesion and tissue integration. These features of IPNs make them appropriate for biomedical applications such as drug delivery vehicles, injectable hydrogels, and regenerative scaffolds.

## 4. Functional Chitosan-Based IPNs

Functional chitosan-based interpenetrating polymer networks have attracted significant attention for combining the intrinsic bioactivity of chitosan with the enhanced physicochemical performance afforded by network interpenetration. Thambiliyagodage et al. [54] reviewed how, through the processes of grafting, crosslinking, composite, s-IPN, or IPN formation, chitosan can be functionalized to introduce certain biological and physicochemical properties, such as an enhanced adsorption capacity, pH-responsiveness and antibacterial activity. These modifications promote the formation of stable and functional network structures with improved control over material performance. Natural polymer-based hydrogel systems comprising chitosan exhibit a tunable water uptake and controlled drug release due to the presence of hydrophilic functional groups and interconnected polymer networks, which enhance transport properties and biological compatibility. Furthermore, Chicea [55] reported that chitosan-based composite and IPN scaffold systems had improved bioactivity, cell adhesion, and tissue regeneration potential. The incorporation of polymeric or inorganic components into the chitosan networks improves pore structure, nutrient transport, and cellular interactions, thereby promoting osteoblast development, collagen expression and tissue integration.

### 4.1. Types of Polymer Combination

#### 4.1.1. Chitosan–Synthetic Polymers IPNs

Chitosan–synthetic polymers IPNs have been extensively studied to improve the physicochemical and biological performance of pure chitosan for biomedical applications. Bhattarai and co-workers [56] prepared chitosan–polyethylene glycol (PEG) hydrogels and demonstrated higher water absorption, improved biocompatibility and sustainable drug release behaviour, emphasizing its applicability for localized drug delivery and tissue engineering applications. Furthermore, Dash et al. [23] found that incorporating synthetic polymers such as PEG (polyethylene glycol), PVA (polyvinyl alcohol) and PAA (polyacrylic acid) into chitosan matrices improves structural stability and degradation behaviour, thereby increasing their biomedical applicability. Berger et al. [57] also described the structural interactions and network formation mechanisms in chitosan hydrogels, which serve as the basis for developing hybrid chitosan–synthetic polymer systems with enhanced functional performance. These systems are consistently reported to provide higher mechanical stability and improved control over swelling and diffusion compared to pristine chitosan.

#### 4.1.2. Chitosan–Biopolymers IPNs

Chitosan biopolymer interpenetrating polymer networks are intensively investigated to increase the biological functionality, biodegradability and physicochemical stability of chitosan by integrating it with naturally derived polymers such as alginate, gelatin, collagen, and hyaluronic acid. Mi et al. [58] prepared chitosan–alginate polyelectrolyte network membranes by ionic complexation between the protonated amino groups of chitosan and the carboxyl groups of alginate, demonstrating enhanced membrane stability, controlled permeability for drug delivery and wound dressing applications. Similarly, Mao et al. [59] synthesized chitosan–gelatin network scaffolds and found that cell adhesion, biodegradability, and structural integrity were improved due to interactions between chitosan and protein-based polymer chains, thereby allowing their use in tissue engineering. In addition, Chang et al. [60] prepared chitosan–hyaluronic acid composite hydrogels and showed that these hydrogels possessed improved biocompatibility, moisture retention, and cellular interaction properties suitable for wound healing and regenerative medicine applications. These studies reveal that integrating chitosan with natural biopolymers results in multifunctional IPN systems with improved biodegradability, biological responsiveness, and structural stability, making them ideal for biomedical and environmental applications. Such systems offer excellent biological compatibility, although their mechanical strength is generally lower compared to synthetic polymer-based IPNs.

#### 4.1.3. Hybrid IPNs with Inorganic Components

Hybrid interpenetrating polymer networks incorporating inorganic components are a significant technique for overcoming the mechanical stability, bioactivity, and functional performance limitations of purely polymeric systems. Wonnie Ma et al. [61] synthesized chitosan–silica hybrid materials via an ionotropic gelation technique, demonstrating enhanced thermal stability and sustainable drug release behaviour due to the formation of a rigid inorganic framework within the polymer matrix. Similarly, Wang et al. [62] prepared chitosan–hydroxyapatite composite scaffolds and showed that the presence of a bioactive ceramic phase considerably increases osteoconductivity and bone regeneration capacity, emphasizing their potential use in bone tissue engineering. Chitosan–graphene oxide hybrid systems demonstrate improved mechanical properties and enhanced cellular responsiveness, resulting from strong interactions between the polymer chains and the nanosheets, as reported by Depan et al. [63]. Furthermore, the incorporation of metallic nanoparticles into chitosan matrices results in additional biological functionality, as Qi et al. [64] reported that a chitosan–silver nanoparticle hybrid possesses strong antimicrobial activity, which is suitable for wound dressing and biomedical coating applications. The incorporation of inorganic or nanomaterials has been shown to significantly enhance adsorption behaviour and structural reinforcement.

#### 4.1.4. Comparative Analysis of Polymer Combinations

A comparison of several chitosan-based interpenetrating polymer network (IPN) systems demonstrates that their effectiveness is highly reliant on the secondary polymer. Chitosan–synthetic polymer IPNs, such as those including poly(vinyl alcohol), poly(acrylic acid) or polyacrylamide, often have higher mechanical strength, structural stability and controlled swelling behaviour due to the formation of dense and well-integrated networks. When compared to pure chitosan, these properties lead to a more lasting and more regulated drug release. In contrast, chitosan–biopolymer IPNs made from natural polymers such as alginate, gelatin or hyaluronic acid have excellent biocompatibility, biodegradability and bioactivity, making them ideal for biomedical applications. However, their mechanical strength and long-term stability are frequently lower due to weaker intermolecular interactions. To illustrate chitosan/polyacrylic acid based IPN hydrogels reinforced with ionic crosslinkers have demonstrated tensile strengths in the range of 0.96–2.56 MPa whereas chitosan–biopolymer networks such as those based on gelatin or alginate, typically exhibit tensile strengths below 1 MPa under fully hydrated physiological conditions [65]. Hybrid IPN systems that include inorganic components or nanomaterials improve adsorption performance and mechanical reinforcement by increasing surface area and providing additional active binding sites but they may pose synthesis complexity, dispersion stability and potential toxicity challenges. Hassan and co-workers synthesized a carbonized chitosan–ZnO–Fe_3_O_4_ nanocomposite and reported maximum adsorption capacities of 891.34 mg/g for Ni^2+^, 1269.35 mg/g for Co^2+^ and 1502.67 mg/g for Cu^2+^ values that far exceed the 30–80 mg/g typically reported for pristine chitosan under comparable conditions highlighting the exceptional enhancement in adsorption performance achievabale through hybrid composite formation [66]. Overall, these studies show that no single system is generally ideal and that the choice of polymer combinations should be driven by application-specific criteria such as mechanical strength, adsorption efficiency and biocompatibility.

### 4.2. Functionalization Strategies

#### 4.2.1. Graft Copolymerization

Graft copolymerization is a widely adopted technique in which chitosan is modified by covalently attaching functional moieties or polymer chains onto its backbone, thereby increasing physicochemical stability, solubility, and performance. In this technique, the reactive amino and hydroxyl groups of chitosan serve as active sites for initiating polymer chain formation via free-radical or chemical grafting mechanisms. Gupta and Ravi Kumar [67] synthesized chitosan-g- poly (AAm) copolymers using ceric ammonium nitrate as an initiator, where poly AAm chains were grafted onto the chitosan backbone, resulting in improved swelling behaviour and controlled drug release characteristics. Amphiphilic semi-IPN hydrogels based on acrylic acid (AA) and chitosan (CS) had a maximum equilibrium swelling ratio of 8550% at alkaline pH for a hydrogel with an AA/CS ratio of 1:0.001 compared to the typical swelling ratios of 200–400% observed for pristine chitosan at low pH, demonstrating the remarkable capacity of grafted copolymerization to enhance water absorption in chitosan-based systems [68]. Sashiwa et al. [69] reviewed various chemical grafting approaches for the synthesis of chitosan derivatives, including free radical polymerization, Schiff base formation, carbodiimide-mediated coupling, ring-opening polymerization, etc., at the amino and hydroxyl groups of chitosan. They emphasized that chemical grafting through these approaches improves physicochemical stability, processability, solubility, and functionality compared to bare chitosan. However, while free-radical grafting methods are relatively simple and widely used, they often suffer from limited control over grafting density and chain distribution, leading to heterogeneity in the final material properties. Furthermore, although grafting generally enhances solubility and functional group density, excessive grafting may reduce the availability of active amino groups, potentially affecting adsorption efficiency. These observations highlight that while graft copolymerization is effective in improving chitosan performance, careful optimization of grafting conditions is necessary to balance functionality, uniformity and adsorption behaviour.

#### 4.2.2. Crosslinking

Crosslinking is a widely employed functionalization technique used to modify chitosan, in which polymer chains are physically or chemically interconnected by forming a three-dimensional network through ionic or covalent bonds to improve mechanical strength, structural stability, chemical resistance, swelling behaviour etc. Yi Zhang et al. [70] Synthesized genipin-crosslinked chitosan/gelatin 3D scaffolds for liver tissue engineering, demonstrating that genipin-mediated crosslinking enhanced scaffold porosity, cytocompatibility, and cell proliferation, highlighting its appropriateness as a low-toxicity natural crosslinker for biomedical applications. Uddin and coworkers [71] investigated chitosan hydrogels crosslinked with genipin and disulfide linkages, highlighting that crosslinking density has a substantial impact on drug-loading and unloading kinetics in targeted delivery systems. However, while increasing crosslinking density generally enhances mechanical strength and structural stability, it may simultaneously reduce swelling capacity and limit the diffusion of therapeutic agents indicating a trade-off between stability and transport properties. In contrast, chemical crosslinkers such as glutaraldehyde and glyoxal often provide higher mechanical strength compared to naturally derived agents like genipin but their potential cytotoxicity restricts their use in biomedical applications. Quantitatively, in vitro studies have established that genipin demonstrates acceptable cyctocompatibility at concentrations of approximately 0.1–1% (*w*/*v*) whereas glutaraldehyde exhibits significant cyctotoxic effects at residual concentrations as low as 0.01–0.1% (*w*/*v*) due to its highly reactive aldehyde groups making genipin a considerably safer crosslinking agent for the fabrication ofbiomedical chitosan scaffolds. Ionic crosslinkers such as sodium tripolyphosphate (TPP) offer milder processing conditions and improved biocompatibility. However, they typically result in weaker and less stable networks. These observations suggest that the selection of the crosslinking strategy must be carefully optimized to balance mechanical integrity, biocompatibility and functional performance in chitosan-based systems [72].

#### 4.2.3. Incorporation of Responsive Moieties

Incorporation of stimulus-responsive moieties into chitosan is an important functionalization strategy that allows the polymer to show environment-dependent physicochemical behaviour, notably for biomedical applications. Due to the presence of reactive functional groups, chitosan can be chemically modified with ionizable or stimuli- responsive functionalities that respond to variations in physiological conditions such as pH, temperature, redox potential, etc. Szymanska and Winnicka [73] reported that pH-responsive behaviour in chitosan can be induced by the introduction of derivatives such as carboxymethyl chitosan, which leads to changes in solubility, swelling and drug release under various pH conditions, making them suitable for site-specific drug delivery. Temperature-sensitive chitosan derivatives are typically prepared by grafting thermosensitive polymers such as PNIPAM (Poly N-isopropylacrylamide) or by preparing thermogelling chitosan solutions, which exhibit reversible sol–gel transitions at physiological temperatures and have been extensively studied for injectable biomaterials and tissue engineering applications. Ways et al. [74] in their review explained that thiolated chitosan derivatives undergo disulfide bond cleavage in reducing environments, which leads to increased mucoadhesion, permeability and controlled drug release. These stimuli-responsive moieties greatly enhance the functional performance of chitosan-based biomaterials in biomedical systems by precisely controlling swelling, degradation, and therapeutic release behaviour. A comparative summary of different functionalization strategies, including their structural features, advantages, limitations and biomedical relevance, is presented in Table 1.

Overall, different functionalization strategies offer distinct advantages depending on the intended application. Chemical crosslinking improves structural stability and mechanical strength, whereas grafting enhances functional group density and adsorption performance. In contrast, green crosslinkers and physically crosslinked systems provide better biocompatibility, but they may exhibit lower stability. These observations highlight the trade-off between mechanical strength, biocompatibility and responsiveness in the design of chitosan-based IPNs.

## 5. Synthesis Routes and Fabrication Techniques

### 5.1. Free Radical Polymerisation

Free radical polymerisation is one of the most commonly used synthesis routes for the fabrication of functional chitosan-based materials such as graft copolymers and hydrogels. The process generally involves free radical initiators such as APS (ammonium persulfate), KPS (potassium persulfate), CAN (ceric ammonium nitrate) etc that activate functional groups of chitosan to form radicals. Vakili et al. [28] in their review on chitosan-based adsorbents explained how vinyl monomers, like AA (acrylic acid), MAA (methacrylic acid), AAm (acrylamide) etc, are attached to chitosan via a free-radical graft copolymerisation technique, thereby increasing the functional group density, improving the adsorption capacity and structural stability. Zhang et al. [84] synthesized chitosan-*g*-AA hydrogels using KPS as a free radical initiator and reported enhanced swelling and improved adsorption performance of the grafted chitosan as compared to bare chitosan. Jayakumar and co-workers [85] discussed the fabrication of chitosan-based graft copolymers through free radical polymerisation for biomedical applications, demonstrating sustainable drug loading and release behaviour due to covalent graft formation. Free radical polymerisation offers a flexible route for tailoring the structural and functional properties of chitosan, achieved through the controlled grafting of functional monomers and the fabrication of advanced polymeric networks.

### 5.2. In Situ Polymerisation

In situ polymerisation is a fabrication method in which polymer formation takes place directly within the chitosan matrix or solution, resulting in the formation of hybrid materials that are structurally integrated with improved homogeneity and enhanced functional performance. Ferri et al. [86] synthesized hybrid nanoparticles of chitosan and poly MAGG (Methacryloylglycylglycine) via in situ polymerisation, which enabled better control over the size distribution and structural stability of the hybrid system. Their study revealed that polymer growth within the chitosan matrix promotes uniform network formation and enhances the functional properties of the material. Similarly, Moreno-Serna and co-workers [87] reviewed different types of in situ-prepared chitosan-based hybrid nanocomposites and reported that polymerisation or formation of functional components within the chitosan results in a homogeneous distribution, improved interfacial compatibility, and increased catalytic and photocatalytic activities of the hybrid materials. The authors highlighted that in situ synthesis leads to strong polymer-phase interactions and structural integration, which are crucial for improving the functionality of chitosan-based systems.

### 5.3. Physical vs. Chemical Crosslinking

Crosslinking can be classified into physical and chemical crosslinking, based on the nature of the interactions that lead to network formation. Pita-Lopez et al. [88] conducted a deep review of physically crosslinked chitosan hydrogels, discussing that the hydrogel networks are formed through non-covalent interactions, such as hydrogen bonding and ionic interactions, without the use of any chemical crosslinking agents. Their study demonstrated the potential of these hydrogels as biocompatible scaffolds that support cell adhesion, proliferation and tissue growth in tissue engineering applications. In contrast, Muzzarelli and co-workers [89] focused on genipin-crosslinked chitosan gels and scaffolds, in which genipin was used as a natural crosslinking agent to improve the structural stability, mechanical strength, and low cytotoxicity of the hydrogels. Furthermore, Rodriguez-Felix et al. [90] prepared chemically crosslinked chitosan hydrogels using L-glutamic acid and assessed their potential for drug delivery applications. Their work involved hydrogel synthesis, structural characterization, swelling behaviour analysis and drug release studies, showing that the prepared hydrogels had controlled drug release characteristics.

### 5.4. Green and Sustainable Synthesis Approaches

Green and sustainable synthesis of chitosan-based polymers is widely used technique, which is investigated using eco-friendly crosslinkers, plant-mediated methods and energy-efficient processing techniques. Fwu-Long Mi and co-workers [91] investigated genipin-crosslinked chitosan networks and showed that genipin functions as a naturally derived crosslinker that forms stable chitosan networks with less cytotoxicity and better biocompatibility than traditional chemical crosslinkers, promoting the formation of safer biomedical materials. Enzymatic modification has also been investigated as a sustainable strategy, in addition to natural crosslinkers. Regarding chitosan derivatives, Sashiwa and Aiba [69] explained moderate chemical modification and enzyme-mediated functionalization pathways emphasizing environmentally friendly reaction conditions and reduced chemical waste during synthesis.

**Figure 3:**
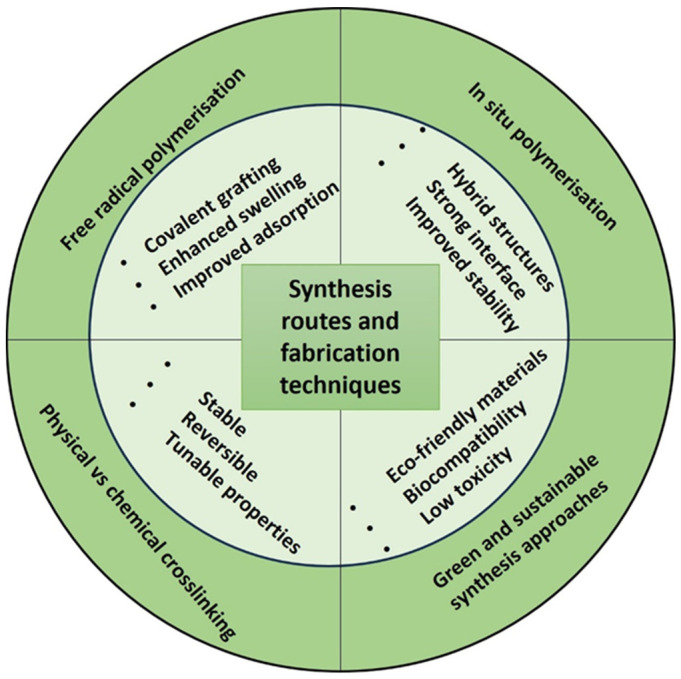
Various synthesis routes and fabrication techniques.

As shown in Figure 3, different synthesis approaches, such as free radical polymerization, in situ polymerization and crosslinking methods, contribute to the formation of interpenetrating polymer networks with distinct structural and functional characteristics.

Despite the efficiency of multiple synthesis techniques, scalability and industrial feasibility are still the key factors. Because of their simplicity and compatibility with traditional processing techniques, free radical polymerisation and in situ polymerisation can be adopted for large-scale manufacturing. However, precise control of reaction parameters, such as initiator concentration and temperature, is required to ensure repeatability. Chemical crosslinking techniques provide strong, durable network formation, but they may be limited by the use of potentially hazardous crosslinking agents that must be carefully removed or replaced with safer alternatives in biomedical applications. Green synthesis techniques, such as the utilization of natural crosslinkers and enzyme-mediated procedures, provide ecologically benign and biocompatible alternatives; however their scalability and reaction efficiency may be restricted. Therefore, future development should focus on optimizing synthesis routes that balance performance, safety, cost-effectiveness and industrial scalability.

## 6. Super-Adsorbent Behaviour of Chitosan IPNs

**Figure 4:**
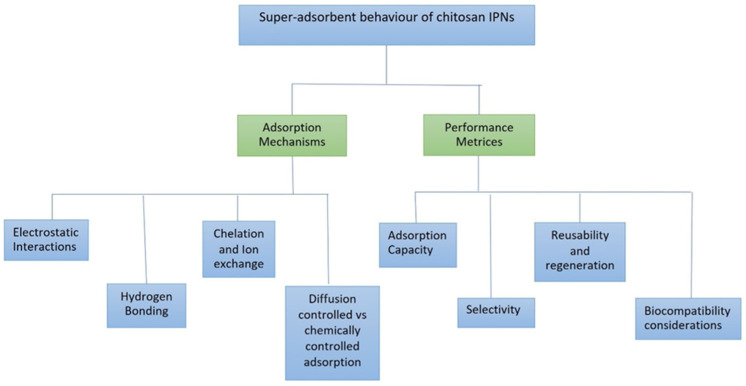
Super-adsorbent behaviour of chitosan-based IPNs.

Interpenetrating polymer networks based on chitosan demonstrate super-adsorbent behaviour due to the formation of a mechanically stable, highly swollen three-dimensional network structure and the synergistic integration of multiple functional groups. As discussed in Section 2, the adsorption behaviour of chitosan is governed by its inherent functional groups, which enable multiple interaction mechanisms with target molecules. Pristine chitosan has limitations, including poor stability under acidic conditions and limited reusability. Pristine chitosan typically achieves adsorption capacities in the range of 30–80 mg/g for heavy metal ions, and 50–150 mg/g for organic dyes, whereas crosslinked and IPN-modified chitosan systems demonstrate capacities of 150–600 mg/g, and advanced hybrid nanocomposite formulations can exceed 800 mg/g under optimized conditions, reflecting the progressive performance enhancement achievable through structural modification [92]. The incorporation of a secondary interpenetrating network enhances porosity and swelling behaviour while maintaining the accessibility of active sites, thereby improving adsorption performance. Crini [93] reviewed how chelating amino groups primarily control chitosan’s adsorption performance towards heavy metals, and structural alteration or network strengthening greatly increases adsorption capacity and reusability. According to Wan Ngah and Hanafiah [94], crosslinking and chemical modification of chitosan improve adsorption effectiveness by improving stability and avoiding dissolution in acidic solutions, allowing for multiple adsorption–desorption cycles. The interpenetrated secondary network in IPN systems promotes diffusion routes and porosity, facilitating the mass transfer of adsorbates to internal binding sites. The three-dimensional crosslinked design of super-adsorbent chitosan IPNs permits high water absorption, resulting in enlarged channels that enhance pollutant diffusion into the matrix. The cooperative impact of chemical functionality and network morphology results in a high adsorption capacity, selectivity towards certain ions, efficient kinetics and superior recyclability. A schematic representation of the adsorption mechanism and network-assisted diffusion in chitosan-based IPNs is illustrated in Figure 4. The term “super-adsorbent” in the context of chitosan-based IPNs refers to their ability to have much increased adsorption capacity, better swelling behaviour and improved structural stability when compared to pure chitosan and traditional adsorbents. This improvement results from the synergistic combination of several functional groups, enhanced porosity and interconnected network frameworks that allow for efficient mass transfer and access to active binding sites. Furthermore, the inclusion of a secondary interpenetrating network inhibits structural collapse and allows for several adsorption–desorption cycles, increasing reusability and overall performance.

### 6.1. Adsorption Mechanisms

The adsorption efficiency of chitosan-based interpenetrating polymer networks is improved by the synergistic interaction of functional groups, network design and physicochemical characteristics. Maximizing adsorbent performance and design in environmental and biomedical contexts requires an understanding of the underlying processes.

#### 6.1.1. Electrostatic Interactions

Electrostatic interactions between adsorbent sites and adsorbate ions in solution are primarily influenced by chitosan’s protonated amino groups, which interact with anionic species. A recent review on chitosan-derived adsorbents demonstrated that electrostatic attraction is one of the key processes governing metal ion and dye adsorption, particularly when functional groups on chitosan interact with charged species in solution. The degree of electrostatic adsorption is highly dependent on the solution pH, because it determines the degree of protonation of the chitosan amines and the charge state of the pollutant species [95].

#### 6.1.2. Hydrogen Bonding

Hydrogen bonding plays an important role in the adsorption of uncharged polar molecules and some organic contaminants, where chitosan’s functional groups serve as donors or acceptors. Hydrogen bonding between the hydroxyl and amino groups of chitosan and the electronegative atoms of adsorbates has been extensively studied in modified chitosan composites, notably in dye removal systems where functional group interactions are validated by spectroscopic shifts. Adsorption experiments on chitosan composite cryogels reveal that hydrogen bonding, in conjunction with other interactions (such as electrostatics), helps to stabilize bound molecules inside the polymer matrix [96].

#### 6.1.3. Chelation and Ion Exchange

Chelation is the selective coordination of metal ions with several donor atoms on an adsorbent, resulting in stable metal–ligand complexes, whereas ion exchange is the displacement of mobile ions on the adsorbent surface by target ions in solution. A mechanistic review of crosslinked chitosan graft copolymer beads revealed that the adsorption of Cu (II) and Cd (II) ions mainly involves chelation with -NH_2_, -OH and C=O groups alongside electrostatic attractions and ion-exchange processes, as validated by XPS (X-ray photoelectron spectroscopy) analysis. This reveals that for metal ions, chelation/complexation and ion exchange coexist with electrostatic processes in chitosan-based adsorbents [97].

#### 6.1.4. Diffusion Controlled vs. Chemically Controlled Adsorption

In adsorption kinetics, the rate of pollutant absorption can be determined by mass movement (diffusion) or chemical interaction rate at the active sites. Adsorption kinetic investigations in chitosan composites show that the uptake process frequently displays multi-stage diffusion behaviour with initial boundary layer diffusion and intra-particle transport taking precedence, followed by chemical surface contact. Studies using the Weber–Morris intra-particle diffusion model revealed different linear regions corresponding to external surface adsorption and pore diffusion, indicating that diffusion processes contribute significantly to the overall adsorption behaviour, although chemisorption at functional groups also plays a role.

Table 2 summarizes the comparative adsorption performance trends observed in different chitosan-based systems. It is evident that IPN-based structures exhibit enhanced adsorption capacity, faster kinetics, and improved reusability compared to pristine chitosan due to their interconnected network architecture and higher availability of functional binding sites.

### 6.2. Performance Metrics

The efficacy of chitosan-based adsorbents is typically assessed using several essential metrics, including adsorption capacity, selectivity, reusability via regeneration cycles, and biocompatibility for biomedical and environmental applications. These parameters determine the adsorbent materials’ efficiency, practicality, and long-term applications in real-world systems. These performance parameters are substantially influenced by structural properties such as surface functional groups, porosity, crosslinking density and polymer composition [98].

#### 6.2.1. Adsorption Capacity

Adsorption capacity (mg/g) refers to the maximum amount of adsorbate that may be retained per unit mass of adsorbent. This value indicates the number of active binding sites and the affinity between the adsorbent surface and the adsorbent molecules. Wang et al. [99] observed that modifications such as grafting, crosslinking or incorporating magnetic materials considerably improve chitosan’s adsorption performance by increasing the surface area, enhancing amino group accessibility and enabling separation after adsorption. Similarly, experimental investigations have indicated that an increased pollutant concentration enhances the adsorption capacity due to a greater driving force for mass transfer from the solution to the adsorbent surface. A chitosan-based magnetic composite exhibited an adsorption capacity of 294.12 mg/g for Cr (VI) under optimized conditions, indicating the strong sorption potential of the modified chitosan material [99]. For comparison, pristine chitosan under similar experimental conditions typically achieves adsorption capacities of only 30–80 mg/g for heavy metal ions such as Cu (II) and Pb (II) clearly reflecting the significant limitation of unmodified chitosan as an adsorbent material. A magnetic xanthate modified chitosan/polyacrylic acid hydrogels had adsorption capacities of 206 mg/g for Cu (II), 178 mg/g for Cd (II), 168 mg/g for Pb (II) and 140 mg/g for Co (II). This represents an increase of 21–82% over the unmodified magnetic chitosan/polyacrylic acid control tested under identical conditions, and confirming the critical role of surface functionalization in improving adsorption performance [65,100].

#### 6.2.2. Selectivity

Selectivity refers to an adsorbent’s capacity to preferentially bind a particular contaminant in the presence of competing ions or molecules. Selectivity in chitosan-based systems is due to the coordination capability of the amino and hydroxyl groups, which can interact preferentially with specific metal ions or organic molecules based on charge density and coordination chemistry. Research on chitosan-based biosorbents has revealed that selective adsorption may occur even in solutions containing competing ions because of variations in ionic radius, hydration energy and coordination behaviour. Investigations on chitosan biosorption systems indicated the preferential adsorption of nickel ions over competing ions such as calcium and chromium demonstrating that chitosan can show high selectivity toward particular metal ions in complicated aqueous systems [101].

#### 6.2.3. Reusability and Regeneration

Reusability and regeneration are significant performance metrics that indicate the economic viability and sustainability of adsorption systems. Wan Ngah et al. [102] reported that adsorbed pollutants on chitosan-based materials can be successfully desorbed using acidic or chelating solutions, allowing the adsorbent to be reused in several adsorption–desorption cycles. Furthermore, the authors showed that regenerated chitosan adsorbents may retain a significant amount of their adsorption capacity after multiple cycles, highlighting their potential for use in practical wastewater treatment applications. More specifically, crosslinked and unmodified chitosan systems have been reported to retain 80–90% of their initial adsorption capacity over 5–10 adsorption–desorption cycles when regenerated using dilute acidic or chelating solutions, whereas unmodified chitosan typically undergoes significant capacity loss after only 3–5 regeneration cycles owing to structural degradation under repeated acidic treatment conditions.

#### 6.2.4. Biocompatibility Considerations

Biocompatibility is a critical factor when using chitosan-based materials in biomedical or environmentally sensitive applications. Kumar et al. [103] reviewed chitosan’s biological characteristics and stated that it has low toxicity, biodegradability, and high compatibility with biological tissues, making it appropriate for biomedical applications such as drug delivery and wound healing. Furthermore, Muzzarelli [104] stated that chitosan’s natural origin and biological activity contribute to its safe interaction with living systems, making it suitable for use in biomedical materials and environmentally friendly adsorption systems.

## 7. Biomedical Applications

### 7.1. Drug Delivery and Controlled Release

**Figure 5:**
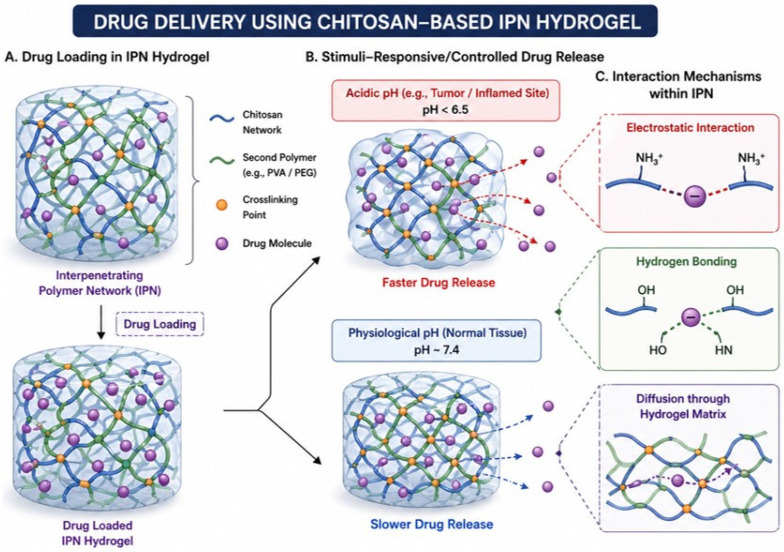
Schematic representation of stimuli-responsive drug delivery using chitosan-based IPNs.

Controlled drug delivery technologies have been widely developed to address the drawbacks of traditional drug administration methods, such as quick drug degradation, non-specific distribution, and variable plasma concentrations. Polymeric biomaterials capable of encapsulating medicinal agents and controlling their release have become more relevant in pharmaceutical research. Chitosan-based materials have gained a lot of interest for drug delivery applications because of their biodegradability, biocompatibility, and ability to form nanoparticles, microspheres, and hydrogel matrices. Calvo et al. [105] synthesized chitosan–polyethylene oxide nanoparticles using an ionic gelation process and proved their capacity to encapsulate proteins as model therapeutic molecules, demonstrating the potential of chitosan nanoparticles as carriers for regulated drug delivery systems. The advantage of the IPN architecture over simple chitosan matrices in drug delivery applications has been quantitively demonstrated by some authors who prepared chitosan/poly (2-hydroxyethyl methacrylate) (CS/p (HEMA)) IPN hydrogel films and reported maximum in vitro drug release rates of 76.0% for doxorubicin and 75.5% for curcumin within 48 h at pH 5.5 and 7.4 respectively. The same study further reported that CS/p(HEMA) IPN films exhibited a swelling ratio of 240% considerably higher than 110% recorded for chitosan/poly (hydroxypropyl methacrylate (CS/p(HPMA)) films under identical conditions, demonstrating that the choice of interpenetrating polymer component directly governs both the swelling and drug release kinetics of the IPN system [106]. In another study, Ilium [107] explored the potential of chitosan-based delivery systems for mucosal drug administration, emphasizing that chitosan’s mucoadhesive nature increases drug residence duration at absorption sites and improves drug bioavailability. These methods are very effective for delivering peptides, proteins, and poorly soluble drugs. Because of these properties, chitosan-derived carriers have been extensively studied for targeted and controlled release applications with enhanced therapeutic efficacy and less systemic adverse effects than traditional drug formulations. Figure 5 shows a schematic illustration of this controlled drug delivery method.

### 7.2. Detoxification and Bio-Separation

**Figure 6:**
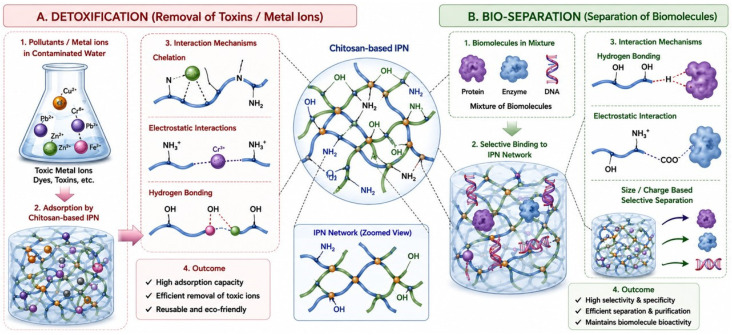
Schematic representation of detoxification and bio-separation system using chitosan-based IPNs.

Effective removal and separation of hazardous chemicals and biomolecules are critical procedures in biomedical therapies and biotechnological purification systems. Materials comprising functional groups that may selectively interact with biomolecules are very advantageous in these applications. Chitosan is intensively studied for detoxification and bio-separation because its polymer structure contains several primary amine groups capable of binding ions and biological molecules via electrostatic attraction, chelation and hydrogen bonding interactions. A schematic representation of the detoxification and bio-separation system using chitosan-based IPNs is depicted in Figure 6. Guibal [25] examined the sorption performance of chitosan and observed that the polymer has a high affinity for metal ions and organic pollutants due to the coordination ability of amino groups present along the polymer backbone. These interactions allow for the generation of stable complexes with hazardous ions, making chitosan-based materials appropriate for detoxification applications. Furthermore, Sashiwa and Aiba [69] reviewed chemical modifications of chitosan and demonstrated that functionalized chitosan derivatives can be synthesized to selectively bind proteins, enzymes, and nucleic acids. These modified matrices have been used as separation platforms in biotechnology to purify biological molecules. The combination of specific binding properties, chemical tunability, and biocompatibility makes chitosan-derived materials promising candidates for detoxification therapy, biomolecule purification, and bio-separation technologies in biomedical fields.

### 7.3. Wound Healing and Tissue Engineering

**Figure 7:**
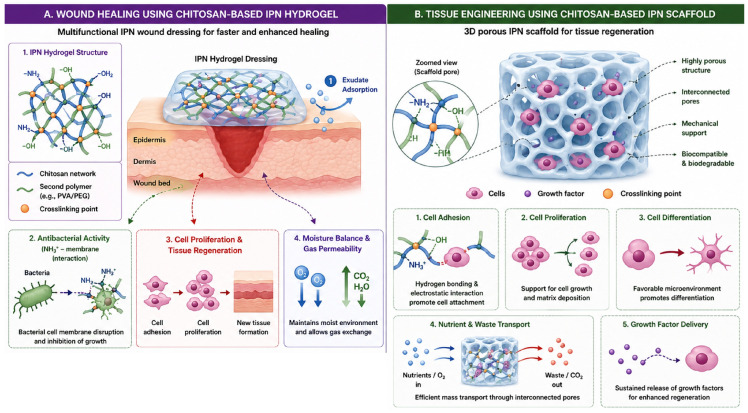
Schematic representation of wound healing and tissue engineering using chitosan-based IPNs.

Biomaterials used in wound healing and tissue engineering must promote cellular development while also protecting the wounded region from microbial infections. Natural polymers have gained more attention in this field because they are structurally similar to biological tissues and show good biocompatibility. Chitosan is regarded as an excellent biomaterial for wound treatment due to its antibacterial and antimicrobial properties, its ability to stop bleeding and to stimulate tissue regeneration. The role of chitosan in wound healing and tissue regeneration is schematically shown in Figure 7. Singh et al. [108] observed that chitosan-based wound dressings improve healing by promoting platelet aggregation, activating macrophages and increasing fibroblast proliferation, all of which led to collagen deposition and the formation of granulation tissue. The authors also explained how chitosan hydrogels, films, and porous sponges maintain a moist wound environment and possess intrinsic antibacterial properties, accelerating wound closure and reducing the risk of infection. In quantitative terms, a chitosan hydrogel wound dressing incorporating silver nanoparticles (AgNPs) prepared by grafting chitosan with poly (N-isopropyl acrylamide) and crosslinking with a PVA/PVP blend demonstrated a swelling ratio of 358.86 ±23.56% and a degradation ratio of 86.02 ± 2.82% over 21 days, confirming its capacity to maintain a sustained moist wound environment throughout the healing process [109]. Furthermore, in an in vivo study using an ischemic rabbit ear wound model, a chitosan–hyaluronic acid–-glycerophosphate in situ-forming hydrogel significantly accelerated wound closure, reducing the mean wound area from 81.8% in the untreated control group to 51.2% by day 9, with histological analysis further confirming enhanced re-epithelialization, collagen maturation and a reduced inflammatory response in the treated group [110]. Similarly, Pramanik et al. [111] emphasized the increasing use of chitosan-based biomaterials in tissue engineering. Their findings showed that chitosan scaffolds, nanofibers, and composite hydrogels may imitate the structural and functional properties of the extracellular matrix, allowing for effective cell adhesion, proliferation, and differentiation. The authors emphasized that chitosan’s controllable physicochemical features allow for the formation of three-dimensional scaffolds with regulated porosity and mechanical stability, which are critical for tissue regeneration. As a result, chitosan-based biomaterials are increasingly being investigated for the repair and regeneration of skin, bone, cartilage, and other injured tissues, indicating great potential in improved wound healing treatments and tissue engineering methodologies.

### 7.4. Biosensing and Diagnostic Applications

**Figure 8:**
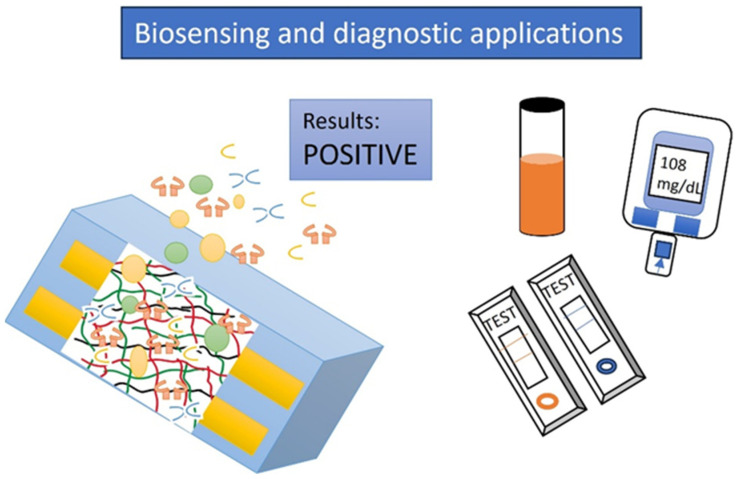
Schematic representation of biosensing and diagnostic applications using chitosan-based IPNs.

Biosensors are analytical devices that use a biological recognition element and a signal transducer to detect particular chemical or biological analytes. The creation of efficient biosensing platforms involves using materials that can immobilize biomolecules while retaining their biological activity and enabling effective signal transduction. Chitosan has been widely used in biosensor manufacturing because of its superior film-forming ability, biocompatibility, and amino functional groups that enable enzyme immobilization. A schematic diagram of the chitosan-based biosensing mechanism is presented in Figure 8. Bocchetta and co-workers [112] reviewed how chitosan can form stable thin coatings on electrode surfaces and creates an ideal environment for immobilizing enzymes, antibodies, and nucleic acids. The authors emphasized that these properties increase the durability and sensitivity of electrochemical biosensors used to detect biologically relevant analytes. Krajewska et al. [113] explained how the amino groups in chitosan enable covalent or electrostatic interactions with biomolecules, allowing for the rapid manufacturing of biosensing platforms. The study found that chitosan-based matrices have been effectively used in biosensors to detect glucose, cholesterol, DNA fragments, and other diagnostic indicators. Furthermore, the integration of chitosan with metal or carbon nanomaterials has been demonstrated to enhance electron transport and sensor sensitivity. As a result, chitosan-based biosensing devices are rapidly being investigated for use in environmental monitoring, clinical diagnostics, and biological analysis.

## 8. Comparison with Other Biomedical Adsorbents

The development of effective adsorbent materials for biomedical purification, detoxification and environmental remediation has gained a lot of attention in recent years. Various types of adsorbents, such as activated carbon, natural polysaccharide-based materials, inorganic minerals, and synthetic polymeric adsorbents, have been widely studied for the removal of harmful pollutants and biologically relevant molecules. Chitosan-based adsorbents have emerged as potential candidates due to their biocompatibility, biodegradability and the presence of reactive functional groups that enable the adsorption processes.

Activated carbon has been recognized as one of the most effective adsorbents due to its large surface area and well-developed porous structure typically exhibiting adsorption capacities in the range of 300–1000 mg/g for various organic contaminants. The microporous network of activated carbon has various adsorption sites capable of binding organic contaminants, dyes and pharmaceuticals. Nonetheless, despite its remarkable adsorption capability, activated carbon has several limitations, especially in biomedical and environmental applications. These include relatively high production costs, complicated regeneration procedures and the possibility of decreased adsorption efficacy with repeated usage. Quesada et al. [114] examined composite adsorbents based on activated carbon immobilized within natural polymer matrices and found that combining activated carbon with biopolymers like chitosan or alginate can significantly improve structural stability and facilitate separation after adsorption. In comparison, chitosan-based materials generally exhibit adsorption capacities in the range of 50–300 mg/g which can be further enhanced to 150–600 mg/g in modified systems such as crosslinked networks or interpenetrating polymer networks (IPNs). The structural basis for this improvement is directly reflected in surface area measurements: pristine unmodified chitosan beads exhibit a BET surface area of only 0.708 m^2^/g whereas chitosan beads modified with FeO/TiO_2_ nanoparticles via ionic crosslinking achieve a surface area of 27.13 m^2^/g and this 38 fold increase in surface area corresponds to improved adsorption capacity rising from 29.8 mg/g for unmodified chitosan to 33.1 mg/g for the composite for naphthalene adsorption under the same experimental conditions. Benettayeb and co-workers [115] examined various modifications of alginate and chitosan adsorbents and concluded that chitosan has a higher adsorption capacity for multiple contaminants due to the strong coordination ability of its amino groups. Furthermore, the authors observed that chitosan-based materials are generally more versatile in adsorption mechanisms since their chemical structure can be easily modified to incorporate new binding sites. However, pristine chitosan often exhibits lower mechanical stability and limited reusability (typically 3–5 cycles) compared to activated carbon. Inorganic adsorbents such as zeolites are also widely utilized in adsorption processes due to their well-defined crystalline structures and strong ion-exchange capacity with reported adsorption capacities typically ranging from 100 to 400 mg/g. Zeolites have a microporous aluminosilicate structure that allows for selective adsorption of certain ions and compounds. These are very effective in removing particularly ammonium ions and heavy metals from aqueous solutions, but their adsorption ability is limited to ion-exchange processes and usually has a lesser affinity for organic molecules than polymer-based adsorbents. Synthetic polymeric adsorbents have also been produced to enhance the adsorption efficiency (typically 200–800 mg/g) and selectivity, but many of these are derived from non-renewable resources and have limited biodegradability, raising concerns about environmental sustainability. In contrast, chitosan-based adsorbents combine biodegradability, several functional groups, chemical tunability, and strong interaction mechanisms, allowing for the effective adsorption of a wide range of contaminants. Through suitable chemical modification and composite formation, chitosan materials can overcome their limitations and emerge as potential adsorbents for biomedical and environmental applications.

## 9. Challenges and Limitations

Despite substantial advances in the development of chitosan-based materials and interpenetrating polymer networks, various challenges still limit their large-scale implementation in biomedical and adsorption applications. One of the main challenges is the scalability and reproducibility of synthesis procedures. Many chitosan-based systems are developed under controlled laboratory conditions using specific crosslinking agents, solvents or nanomaterials, which might be difficult to reproduce on a large scale. Variations in the degree of deacetylation, molecular weight and purity of chitosan obtained from various sources can further influence the physicochemical characteristics of the resultant materials, causing performance variability. Another major aspect is the mechanical strength and long-term stability. Although chemical modification and crosslinking strategies can improve structural integrity, native chitosan exhibits limited mechanical strength and reduced stability, which limits its durability during repeated adsorption or biomedical applications. Furthermore, regulatory and toxicity considerations must be carefully addressed, especially when chitosan-based materials are intended for biomedical applications such as drug delivery or implantable scaffolds. The incorporation of crosslinking agents, nanoparticles and synthetic polymers may introduce potential toxicity problems, requiring extensive biocompatibility and safety testing before clinical or commercial implementation. For instance, glutaraldehyde, one of the most widely used chemical crosslinkers, has been reported to exhibit cytotoxic effects even at relatively low residual concentrations (typically >0.01–0.1% *w*/*v*), primarily due to its highly reactive aldehyde groups. Therefore, its use in biomedical systems requires strict control of concentration, as well extensive post-crosslinking washing to remove unreacted residues. In contrast, genipin is considered significantly less toxic, with studies reporting acceptable cytocompatibility at concentrations in the range of ~0.1–1% (*w*/*v*) depending on the system. Similarly, ionic crosslinkers such as sodium tripolyphosphate (TPP) are widely regarded as safe due to their low toxicity and mild reaction conditions. In vivo studies further support these observations, where genipin-crosslinked chitosan scaffolds have demonstrated minimal inflammatory response, good biocompatibility and enhanced tissue integration compared to glutaraldehyde-crosslinked systems. From a regulatory standpoint, glutaraldehyde is approved by the FDA primarily as a disinfectant and sterilizing agent but is not preferred for implantable biomaterials due to toxicity concerns. In contrast, naturally derived crosslinkers such as genipin, as well as enzymatic crosslinking approaches (e.g., transglutaminase) are increasingly explored for biomedical applications due to their improved safety profiles, although full FDA approval for specific applications depends on the final material formulation. Furthermore, cost and processing constraints also remain crucial factors influencing practical applicability. Although chitosan is derived from natural resources, the processing procedures involved in purification, modification and fabrication of advanced composites or IPN systems can raise overall production costs. Therefore, future studies should focus on developing economically feasible, scalable and ecologically friendly manufacturing processes while maintaining the functional performance of chitosan-based materials. The current literature on chitosan-based IPNs has limitations in addition to these typical difficulties. Despite the fact that several functionalization techniques, such as graft copolymerisation, crosslinking and hybrid composite production, have been extensively studied, there is frequently a lack of a direct comparison of their efficacy. For instance, chemical crosslinking greatly increases mechanical strength and stability, but depending on the crosslinking agent employed, it may also minimize swelling behaviour and introduce possible toxicity. Similarly, grafting methods increase the density of functional groups, which improves adsorption capacity, but they may have limited repeatability and variability. Although hybrid nanocomposite systems show better mechanical and adsorption performance, problems with nanoparticle aggregation, synthesis complexity and scalability still need to be addressed.

## 10. Conclusions

Chitosan-based polymeric systems have gained considerable attention in recent years because of their unique physicochemical and biological properties. Chitosan is derived from a natural biopolymer, chitin, and exhibits excellent biocompatibility, biodegradability, and non-toxicity. The presence of amino and hydroxyl functional groups on its chain enables the versatile chemical modifications. These properties have allowed the development of a wide range of advanced materials derived from chitosan, such as graft copolymers, crosslinked networks, interpenetrating networks, and hybrid nanocomposites. These structural modifications greatly enhance the mechanical strength, physicochemical stability and functional performance of chitosan materials as compared to native chitosan. Recent research has shown that chitosan-based materials are extremely versatile in biological and environmental applications. Chitosan based IPNs and hybrid materials provide enhanced adsorption capacity and structural stability by integrating multiple polymeric networks in a synergistic manner. These materials have demonstrated promising performance in drug delivery, detoxification, bio-separation, biosensing, wound healing, tissue engineering and environmental remediation. The adsorption behaviour of chitosan materials is primarily influenced by mechanisms such as electrostatic interactions, hydrogen bonding, ion exchange and chelation with metal ions, which allow for the efficient binding of a wide range of contaminants and biomolecules.

Furthermore, the integration of inorganic nanoparticles or conductive polymers into chitosan matrices has expanded their functionality and application potential. Despite these advancements, several limitations remain that prevent the large-scale utilization of chitosan-based adsorbents and biomaterials. Native chitosan has low mechanical strength, limited stability and a lower surface area than highly porous conventional adsorbents. Although several chemical modification strategies have been developed to address these challenges, further studies are needed to improve material durability, optimize synthesis procedures and enhance regeneration efficiency. In addition, translating chitosan-based systems from laboratory research to clinical and industrial applications remains a significant challenge due to issues related to batch-to-batch variability, lack of standardized synthesis protocols and stringent regulatory requirements for biomedical materials. Therefore, addressing these translational barriers will be essential for the successful commercialization and clinical implementation of chitosan-based technologies.

## 11. Future Perspectives and Research Directions

Although substantial progress has been achieved in the development of chitosan-based materials, further studies are required to fully realize their potential in biomedical and adsorption-related applications. One key area of study is the development of multifunctional and stimuli-responsive chitosan networks. Incorporating pH-, temperature- or redox-responsive moieties into the chitosan matrix allows for regulated drug release, selective adsorption and better responsiveness under physiological conditions. Recent studies have shown that such smart chitosan-based systems can increase the precision and efficiency of biomedical therapies and targeted delivery systems. In addition, future studies should focus on the synthesis of hybrid and nanostructured nanocomposites to improve their functional performance. The incorporation of nanomaterials such as metal nanoparticles, magnetic particles or carbon-based nanostructures can greatly enhance the surface area, adsorption efficiency and mechanical stability, broadening their application in environmental and biomedical systems. Furthermore, the development of green synthesis procedures and scalable production techniques will be crucial for transferring laboratory-scale chitosan materials to practical applications. Future research should also emphasize the standardization of synthesis protocols, including control over parameters such as the degree of deacetylation, molecular weight and crosslinking density to ensure reproducibility and consistent material performance across different studies and industrial production batches. From a clinical perspective, more in vivo studies and long-term biocompatibility assessments are required to evaluate the safety, degradation behaviour and therapeutic efficiency of chitosan-based systems. Addressing these aspects will be critical for regulatory approval and successful clinical translation. Additionally, regulatory considerations must be integrated early in material design, including compliance with guidelines related to toxicity, sterilization and quality control. The identification and use of low-toxicity or FDA-acceptable crosslinking strategies will further support the development of clinically viable chitosan-based biomaterials. Advances in nanotechnology and polymer engineering are expected to enable the development of next-generation chitosan-based materials with increased stability, selectivity, and multifunctionality.

## Figures and Tables

**Table 1:** Comparison of functional chitosan-based IPNs and modification strategies.

Type of System	Functionalization Strategy	Structural Features	Key Advantages	Limitations	Applications	Ref.
Chitosan–Synthetic Polymer IPNs (e.g., CS-PAA, CS-PVA)	Free radical polymerisation, chemical crosslinking	Interpenetrated networks with covalent crosslinks	Improved mechanical strength, enhanced swelling, tuneable properties	Possible toxicity of synthetic components, reduced biodegradability	Drug delivery, adsorption, tissue engineering	[23,75]
Chitosan–Biopolymer IPNs (e.g., CS-alginate, CS-starch)	Ionic + chemical crosslinking	Biocompatible interpenetrating networks	High biodegradability, non-toxicity, eco-friendly nature	Lower mechanical strength, limited stability in harsh conditions	Wound healing, packaging, biomedical scaffolds	[76]
Hybrid IPNs (CS-Fe_3_O_4_, CS-silica)	In situ incorporation, sol–gel, co-precipitation	Organic-inorganic hybrid network	High adsorption capacity, improved thermal/mechanical stability, easy separation	Aggregation of nanoparticles, synthesis complexity	Water treatment, catalysis, biosensing	[77,78]
Graft-Copolymerised Chitosan IPNs	Free radical grafting (KPS, CAN, APS)	Side chains covalently attached to chitosan backbone	Enhanced functionality, improved adsorption sites, better stability	Possible heterogeneity in grafting, process control issues	Adsorption, smart materials, drug delivery	[79]
Chemically Crosslinked Chitosan IPNs	Crosslinking (glutaraldehyde, genipin, citric acid)	Covalent network formation	High structured stability, enhanced swelling, durability	Potential toxicity, reduced flexibility	Tissue engineering, drug delivery, hydrogels	[80]
Physically Crosslinked/Pseudo-IPNs	Hydrogen bonding, ionic interactions	Non-covalent interpenetration	Reversible behaviour, stimuli responsiveness, easy processing	Lower mechanical strength, poor long-term stability	Injectable hydrogels, cell encapsulation, smart delivery	[81]
Stimuli-Responsive Chitosan IPNs	Incorporation of pH/temperature/redox-moieties	Environment-responsive network	Controlled drug release, targeted delivery	Complex synthesis, sensitivity to environmental fluctuations	Drug delivery, biosensors, smart hydrogels	[82]
Nanostructured/Composite Chitosan IPNs	Nanomaterial incorporation (CNTs, metals)	High surface area, multifunctional network	Enhanced adsorption, conductivity, mechanical strength	Cost, aggregation issues, reproducibility challenges	Biosensing, environmental remediation, energy devices	[83]

**Table 2:** Comparison of adsorption behaviour in pristine chitosan and modified chitosan-based systems.

System Type	Adsorption Capacity	Kinetics	Reusability	Key Advantage
Pristine Chitosan	Moderate	Slow	Limited	Natural Functionality
Chitosan IPNs	High	Faster	Improved	3D porosity + diffusion pathways
Crosslinked Chitosan	Moderate-High	Moderate	Good	Resistance to dissolution
Hybrid IPNs	Very High	Fast	Excellent	Surface area + active sites

## Data Availability

No new data were created in this work.

## Abbreviations

The following abbreviations are used in this manuscript:

AA	Acrylic acid
AAm	Acrylamide
APS	Ammonium Persulfate
CAN	Ceric ammonium nitrate
CS	Chitosan
DD	Degree of deacetylation
-*g*-	Grafted
IPNs	Interpenetrating networks
s-IPNs	Semi interpenetrating networks
KPS	Potassium persulfate
MAA	Methacrylic acid
MAGG	Methacryloylglycylglycine
PEG	Polyethylene glycol
PVA	Polyvinyl alcohol
PAA	Polyacrylic acid
PNIPAM	Poly (N-isopropylacrylamide)
TPP	Thiamine Pyrophosphate
XPS	X- ray photoelectron spectroscopy
